# Address

**Published:** 1878-05

**Authors:** Geo. L. Field


### THE DENTAL REGISTER.

Vol. XXXII.] **MAY, 1878.** No. 5.

### ADDRESS.

*Before the Graduating Class of the Dental College of the University of Michigan, March 27th, 1878.*

BY GEO. L. FIELD, D. D. S.

Gentlemen Graduates of the Dental Department of the University of Michigan:—To me has been deputed the very pleasant duty of saying to you, in behalf of the dental profession at large, a few words, both of encouragement and caution. I say pleasant duty for it is a pleasant one. You all have long looked forward to this hour with feelings of joy, yet not unmingled with sorrow. With joy that the long sought goal has at last been reached, a destination only gained after hard labor, steady application, earnest effort and the unwearied devotion of competent teachers. With sorrow that here your little band must be broken up, each to strike out into the wide world alone and each for himself; here ties that have bound you together in sweet companionship must be loosened, possibly to be never again renewed. You take your teacher by the hand and say farewell; the lip may quiver and the eye be dimmed by a tear, but you have at last got your harness on and you are eager for your work. Well, God speed you, I only hope that your highest expectations may be attained and your happiness be made complete.

This is a grand epoch in your life, gentlemen; what a change a few short moments have brought about. I never until this time had much faith in the Darwinian theory, but I must confess that some of my strongest and most fully formed conclusions on this subject have received a pretty severe shaking up; it was but a few moments ago that you entered this hall a poor, insignificant Mr. Tadpole, and now I am of a necessity compelled to hail you as Dr. Frog. What a wonderful transformation in so short a time. Doctor! Does not your heart almost stop its beating, and the cold, clammy perspiration start from every pore as you for the first time are addressed by your title? Swallow that lump in your throat that so seriously interferes with your respiration; put a bold front on the matter, the thing has got to come, and the quicker you adapt yourselves to circumstances the better, you have earned your title honorably. You have complied with all the requirements demanded of you before it was bestowed and now it is yours, and you may wear it proudly. But never in your future career do ought that shall bring the blush to the cheek of either yourself or those that have labored so hard to prepare the mantle that has now fallen upon you. Some few days since I was honored with an invitation from the dental faculty of this school to pay you a visit and inspect some of the results of your labors of the past winter, which invitation I gladly accepted, and it gives me much pleasure to bear testimony to the fact that you have neither been idle, nor have your employed hours been barren of good results. I was not only pleased with what I saw, but I was also surprised in many cases, for I saw much that would have done honor to older heads than yours, and it only proved conclusively to me what thorough and systematic training and study would do.

You have enjoyed advantages that few of us who have long years been treading the paths that you have just entered upon, ever dreamed of, and you have reaped the benefits of our experience gained by harder labor than you have or ever will be required to endure, but do not for one moment think that your labors are over, they have but just begun, and he of you who may be so short sighted as to think that such is the case will certainly come to grief; and in years now far in the future, when he shall have traveled over paths strewn with both flowers and thorns, paths beset with trials and vexations on the one side, and some bright sunny spots on the other, he will look back upon this hour and say, "well, what a little insignificant tadpole I was." If such is not the case, then you may consider yourself much more fortunate than any of your predecessors.

It is but a few years ago, and that within my day, when there were but one or two dental colleges in the United States, and dentistry was but a puny babe, squealing and kicking in its mother's arms. That is, providing dentistry ever had a mother, which matter I am not quite sure about, at any rate it was but a sickly infant, but it grew apace and gathered strength from year to year, under the fostering care of a few earnest fathers, and now the babe has shaken off its swaddling clothes and is making quite a respectable appearance in garments more suitable to mature years, and at last even the medical faculty are ready to take the youth by the hand and say, "Good morning, Doctor," but still the doctor part sticks in their throat. When the boy shall have attained his majority then it will come much easier for them, and they will then be nearer the kingdom, and in the entrance we will pray for them and say, "Forgive them, Father, for they know not what they do."

And now you are about to start out in life for yourselves; the necessary time for incubation has been completed; the shell, as it were, has been broken, and the cocks and hens are clucking and crowing at the results of their labors. You feel cramped and restive from your long confinement, and even with your pin feathers on you are chafing under restraint, so the brood must scatter; you each, no doubt feel quite competent to scratch for yourselves, but don't feel disappointed if you find it hard scratching for a while; it is much easier around the coop than when farther away, and though I would not at this time, when your hearts are overflowing with happiness and everything looks so bright before you, say anything that should dispel your beautiful dream and dampen your ardor, yet in all kindness I would suggest that you do not let your enthusiasm carry you away, take it coolly and do not attempt to crow until you are quite sure that you are of the crowing kind. I have seen some ludicrous mistakes of this kind in a young brood.

A certain amount of self-reliance you will find to be indispensable in life's great battle, and yet it should always be tempered by modesty, though too much of the latter is probably as bad as none at all. I individually have been a sufferer from this cause all my life, you will be expected in time, after the leaven which has been thus far kneaded into your plastic minds, and you have received much more, (which you always will be needing), has had time to work, to give to others as freely as you have received; this we trust you will be always willing and eager to do; give of this bread to those that ask it, but a due regard for modesty will prompt you to look well to it that your cake may not be doughy.

In your new field of labor many temptations will beset your path on every side, you will have abundant opportunities to say unkind things, and to make damaging remarks of others in the same profession that you are following, hoping thereby to benefit yourselves, but let me caution you to never do it, it will as surely rebound upon your own head, as that the hand will be burnt that is thrust into the fire. Such weapons belong in the hands of cowards; when you can not say a kind word, say none at all, yet I would not have you to understand me to counsel the giving the right hand of fellowship to the unprincipled quack and vile pretender, let him be even beneath your notice, never cross swords with such a man. "He that touches pitch will be defiled." In other words be a gentleman, and that will be a sufficient code of ethics to govern you in your practice.

You are now, no doubt, eagerly looking around you for some eligible place in the which to locate for the practice of your chosen profession, and you have all no doubt, determined that nothing short of a large city will ever give you sufficient scope for the display of your unbounded talents, and the bright plumage that must soon take the place of the before mentioned pin feathers, and it does seem a shame that such brightness should be hid under the bushel of a country town. A city practice, by all means gentlemen, "There's millions in it." Yet do not be surprised if you find it hard work to get those millions out of it.

To be sure, all the great guns in the dental profession always live in the city—(I live in the city myself), yet I am compelled to confess that I have seen some of the smaller and less pretentious artillery that did the better execution. Yet whilst I would caution you to look your ground well over before making a decided stand, I still consider this a matter of minor importance as compared with many other things.

In your practice be courteous and gentle with all. There will be times when you go to your chair, that you are feeling irritable, cross and quite unfit for the labor before you. Such things are but natural, and are the results of many causes. Those of you that shall join the great army of benedicts, may be sufferers from too much mother-in-law. This is often the case, but even then, gentlemen, your hearts should be lifted up in gratitude that they come only one at a time, and I have found from personal experience, that walking my chamber half of the night in scanty apparel, with a fretful infant in the one hand and a nursing bottle in the other, was not conducive to a quiet frame of mind, and these trials are undoubtedly in store for you all. So when you from such or other causes shall feel yourself unfitted to do that justice to your patients that you feel you should, quietly dismiss them to a future time, when you may hope to be in a happier frame of mind.

Let your offices be models of neatness and cleanliness. You are all quite conversant with the old adage: "Cleanliness is next to godliness," as a general thing that is undoubtedly so, but in a dental office, I think that cleanliness should take precedence, and if it takes half your yearly income for soap and water in order to gain this result, spend it thus, and spend it freely; you will still have enough to live on, for Geo. Francis Train says that a man may live and grow fat on nine cents a day, and oatmeal porridge will do it, but I trust that you may not be forced to this dire extremity. In the fitting up of .your offices, always have an eye to neatness, and give them as home like and attractive an appearance as your means will allow, if your balance in bank will admit of elegance, well and good, but let everything savor of taste and refinement. Such outward surroundings are but the reflection of an elevated and refined mind.

In the selection of such instruments and appliances as you will require in your practice, purchase none but the best, it will require a little more money in the beginning, but they will prove by far the most economical in the end. A poor instrument is little better than none at all.

Should you deem it advisable through the public press, or by a business card, to call the public's attention to the fact that your services are at their disposal, let modesty govern you to its fullest extent, let such announcement contain nothing more than your name, occupation and place of business, anything more than this savors of quackery, and should be sufficient to lower you in the estimation of those whose good opinion you should most desire.

Don't feel discouraged if business does not come in quite as fast in the beginning as you would wish, remember the old nursery ballad that you learnt in your childhood, "great trees from little acorns grow." Bide your time, don't try to crowd things, don't be over anxious to get rich, riches are sure to come in good time. Who ever heard of a poor dentist who had enjoyed a half dozen years of practice? Especially in a city, dentist's fees are so enormously high you know, at least I take it for granted you do, if not, you will soon be informed of the fact by your patients, even though you charge them only half what your material costs and throw in the labor besides. So with such guarantees you may have little fear of not in a very short time being able to retire to an elegant brown stone front, a tower in one corner, and bay windows in the kitchen, and a most charming mortgage that shall cover the whole establishment. Your tower may prove insecure and fall, your bay windows may open their seams and part from their attachment, but don't feel down hearted gentlemen, your fast friend, the mortgage will stick to you to the bitter end.

And now my friends, I'll not weary you any longer, brevity being the soul of wit, I shall make that my only claim on your indulgence. I have not thought it advisable, even had I been competent to deliver to you either a classical or profound address, the formidable array of scholars with those great store houses of knowledge on their shoulders that now occupy this stage with me would be sufficient to intimidate even a much bolder man than myself, and as "discretion is the better part of valor," I trust none will feel disposed to dispute my bravery, and in taking my leave of you, allow me to thank you for the kind indulgence shown me in this my feeble efforts as an orator, and to welcome you in behalf of those for whom I speak, both to the ranks whose muster roll you now have signed, and also to your hearts, we wish you a long and useful life, and if use to others shall be your end and aim in life, then when the time shall come, which comes to all, when you shall lay down the mortal to take on immortality, then no sting of sorrow and shame shall cast its pall over the records of an ill spent life, peace shall be your end, and your reward shall be sure. "So live, that when thy summons comes, to join the innumerable caravan that moves to the pale realms of shade, where each shall take his chamber in the silent halls of death, thou go not like the quarry slave at night, scourged to his dungeon; but sustained and soothed by an unfaltering trust, approach thy grave like one that "wraps the drapery of his couch about him, and lies down to pleasant dreams."

